# The hidden power of secondary metabolites in plant-fungi interactions and sustainable phytoremediation

**DOI:** 10.3389/fpls.2022.1044896

**Published:** 2022-12-12

**Authors:** Neveen Atta Elhamouly, Omar A. Hewedy, Amr Zaitoon, Angelica Miraples, Omnia T. Elshorbagy, Suzan Hussien, Amira El-Tahan, Deliang Peng

**Affiliations:** ^1^ State Key Laboratory for Biology of Plant Diseases and Insect Pests, Institute of Plant Protection, Chinese Academy of Agricultural Sciences, Beijing, China; ^2^ Department of Botany, Faculty of Agriculture, Menoufia University, Shibin El-Kom, Egypt; ^3^ Department of Plant Agriculture, University of Guelph, Guelph, ON, Canada; ^4^ Department of Food Science, University of Guelph, Guelph, ON, Canada; ^5^ School of Natural and Environmental Sciences, Faculty of Science, Agriculture & Engineering, Newcastle University, Newcastle upon Tyne, United Kingdom; ^6^ Botany Department Faculty of Science, Mansoura University, Mansoura, Egypt; ^7^ Plant Production Department, Arid Lands Cultivation Research Institute, the City of Scientific Research and Technological Applications, City of Scientific Research and Technological Applications (SRTA-City), Borg El Arab, Alexandria, Egypt

**Keywords:** phytoremediation, secondary metabolites, phytopathogenic fungi, plant metabolic response, biotrophic fungi, siderophore, soil mycobiota, Arbuscular mycorrhizal fungi (AMF)

## Abstract

The global environment is dominated by various small exotic substances, known as secondary metabolites, produced by plants and microorganisms. Plants and fungi are particularly plentiful sources of these molecules, whose physiological functions, in many cases, remain a mystery. Fungal secondary metabolites (SM) are a diverse group of substances that exhibit a wide range of chemical properties and generally fall into one of four main family groups: Terpenoids, polyketides, non-ribosomal peptides, or a combination of the latter two. They are incredibly varied in their functions and are often related to the increased fitness of the respective fungus in its environment, often competing with other microbes or interacting with plant species. Several of these metabolites have essential roles in the biological control of plant diseases by various beneficial microorganisms used for crop protection and biofertilization worldwide. Besides direct toxic effects against phytopathogens, natural metabolites can promote root and shoot development and/or disease resistance by activating host systemic defenses. The ability of these microorganisms to synthesize and store biologically active metabolites that are a potent source of novel natural compounds beneficial for agriculture is becoming a top priority for SM fungi research. In this review, we will discuss fungal-plant secondary metabolites with antifungal properties and the role of signaling molecules in induced and acquired systemic resistance activities. Additionally, fungal secondary metabolites mimic plant promotion molecules such as auxins, gibberellins, and abscisic acid, which modulate plant growth under biotic stress. Moreover, we will present a new trend regarding phytoremediation applications using fungal secondary metabolites to achieve sustainable food production and microbial diversity in an eco-friendly environment.

## 1 Introduction

Plants and their associated fungi share a long history of co-evolution. Indeed, beneficial and/or pathogenic relationships with fungi are likely one of the critical traits acquired by the first terrestrial plants (Heckman et al., 2001; Humphreys et al., 2010). The association between fungal microbes (e.g., mycorrhizal, endophytic, saprophytic, and pathogenic microbes) and their host plants have shaped and formed various modifications. Generally, fungi interact with the host plants in different ways. For instance, necrotrophs survive on dead host tissues, biotrophs feed on plant resources without killing the host plant, and hemibiotrophs have an asymptomatic period followed by a necrotrophic stage (Horbach et al., 2011). Interestingly, the beneficial side of soil microbiota is a promising sustainable alternative to improve crop health and productivity. For example, mycorrhizal root colonization positively influenced plant growth and nutrient uptake (e.g., N and P availability). In addition, combining multiple rhizospheric microbes, such as *B. amyloliquefaciens*, *mycorrhizal*, and *Pseudomonas*, significantly varied the plant microbiome structures. As a result, it has positive effects on plant growth, by improving nutrient acquisition and/or water uptake, and the plant nutrient use efficiency (Cozzolino et al., 2021; Chandrasekaran, 2022).

However, biocontrol agents as beneficial microbes or their chemical metabolites are essential in protecting plants against destructive phytopathogenic fungi, even under unfavorable environmental conditions. These chemical molecules, known as Secondary Metabolites (SMs), were defined by (Kossel, 1891) as molecules generated mainly by microbes and plants with a relatively low molecular weight (in most cases, three kDa). Various chemical components used in pharmaceutical and commercial purposes are derived from filamentous endophytic fungi. According to biochemical synthesis, fungal SMs are classified into polyketide, nonribosomal, alkaloids, and terpenes, which have potential ecological and medicinal applications (Sinha et al., 2019; Yu et al., 2021). The adage “necessity is the mother of invention” appears true when we consider plants, despite being nonmotile and simply lacking a highly developed immune system. Plants are not powerless against a wide range of biotic and abiotic stresses; instead, wielding an arsenal of chemicals to deter enemies, fend off pathogens, outcompete competitors, and outperform environmental constraints (Ballhorn et al., 2009; Schäfer and Wink, 2009). Although SMs could not be involved in fundamental cellular operations, they perform essential bio-ecological roles assisting the host organism in adapting to its environmental niche and regulating interactions between species (Perez-Nadales et al., 2014; Scharf et al., 2014). Previously, plant biologists paid little attention to these chemical molecules because they were formerly assumed to be physiologically unimportant. On the other side, organic chemists have studied their chemical structures and characteristics intensively since the 1850s and have a different point of view. It has been suggested that SMs play an active and critical role in possible defensive mechanisms, particularly chemical warfare between plants and pathogens (Croteau et al., 2000).

These molecules have also been identified in plants as herbivore repellents, pollinator attractants, allelopathic agents, toxicity protection, UV-light shielding, and signal transduction. Diverse fungal bioactive secondary metabolites include phytotoxins (SMs secreted by phytopathogenic fungi that attack plants), mycotoxins (SMs produced by fungi that invade crops and cause toxins in humans and other animals), pigments (colored substances with antioxidant properties), and antibiotics (natural products capable of inhibiting or killing microbial competitors) (Demain and Fang, 2000; Keller et al., 2005; Chiang et al., 2009). Additionally, these metabolites play a crucial role in mediating fungal activity, such as sporulation and hyphal extension, while others target plant development and metabolism (Keller et al., 2005). Through this review, we shed light on several topics showing fungal-plant associations and summarize the hidden power of their secondary metabolites to achieve sustainable economic food production under resecure environmental resources.

## 2 Plant secondary metabolites

### 2.1 Hormone production and plant resistance

Plant secondary metabolites are categorized into three groups based on the biosynthetic pathways they are derived from: (1) flavonoids and phenolic compounds, (2) N- containing alkaloids, and (3) terpenoids. These metabolites are essential for plant fitness and survival *via* signaling hormones and attractive substances for pollinators and seed development that modulate plant growth (Wink, 2003). A recent study by (Bean et al., 2022) argued that the cytokinins generated by plant symbiotic *Trichoderma* strains might be exploited to stimulate plant development, affecting the symbiotic fungi’s colonization strategy and regulating the host plant’s phytohormone for improved plant resistance to diseases. In addition, a plant-fungus interaction system was created to study the role of auxin in controlling the growth and maturation of Arabidopsis (*Arabidopsis thaliana*) seedlings concerning infection with *Trichoderma virens* and *Trichoderma atroviride* (Contreras-Cornejo et al., 2009). Another study (Contreras-Cornejo et al., 2015b) measured stomatal aperture and excess water in leaves of Arabidopsis wild-type (WT) seedlings and ABA-insensitive mutants, *abi1-1 and abi2-1*, inoculated with *T. virens* or *T. atroviride*. Compared to untreated seedlings, inoculated Arabidopsis WT seedlings had lower stomatal aperture and water loss. *T. atroviride* is thought to modify root-system architecture by altering mitogen-activated protein kinase 6 activity and ethylene and auxin action (Contreras-Cornejo et al., 2015b).

**Figure 1:**
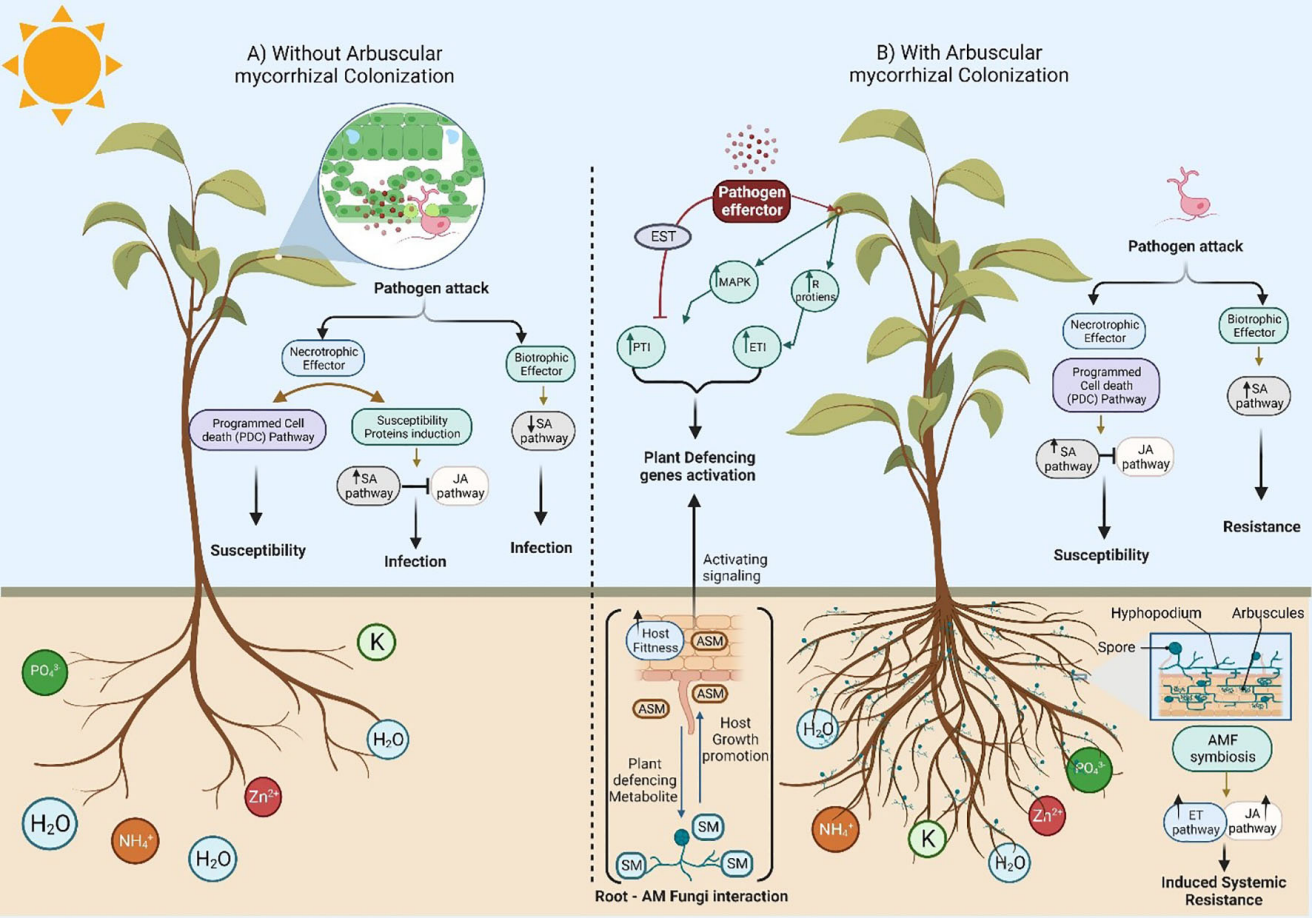
A schematic diagram to show the different plant responses against pathogenic fungi in the absence and presence of Arbuscular mycorrhizal fungi (AMF) colonization. **(A)** Absence of root colonization by AMF causes more damage when compared to mycorrhizal plants due to the development of symptoms in response to necrotrophic and biotrophic pathogens. In addition, host plants with undeveloped root systems have a low ability to uptake nutrients from the soil, leading to plant death by the end. **(B)** A symbiotic relationship between plant roots and Arbuscular mycorrhizal fungi (AMF) significantly alters ecosystems and impacts plant production via plant growth promotion due to improved acquisition of mineral nutrients through the extensive AM fungal hyphal network (mycorrhizosphere) with a massive mycorrhizal network around the root system. Furthermore, host plants can thrive under various abiotic/biotic stresses (including drought, salt, herbivory, temperature, metals, and pathogens) due to the symbiotic localization of Arbuscular mycorrhizal fungi (AMF) via complex signal communications that increase the photosynthetic plant rate. Hence, the release of strigolactones (SLs) as part of the root exudates induces the branching of AMF hyphae to promote mycorrhization. Changes in the root exudate patterns induce changes in the soil microbial community, possibly by attracting antagonists of pathogens. In addition, there are various ways of AMF-induced biotic stress tolerance in plants *via* competition with soil pathogens and nutrients uptake, altered root exudates which support beneficial microbes and suppress phytopathogens in the rhizosphere, AMF colonized roots have little or no space for pathogen entry. Interestingly, a general reduction in the damage and incidence of disease caused by soilborne pathogens was noticed due to defense power from the priming of the plant. The role of the phytohormones (e.g., JA and ET) in the relationship between the host plant and its symbiotic fungi are well known. Phytohormones participate as signaling molecules and improve host plant ISR (Induced Systemic Resistance). In contrast, the development of necrotrophic pathogens in plant–fungal pathogen interaction signals is restricted due to the primed jasmonate-regulated plant defense mechanisms.

Phytohormones such as ethylene (ET) (Soanes et al., 2002), salicylic acid (SA) (Janda and Ruelland, 2015), jasmonic acid (JA) (Wasternack, 2007; Quirino et al., 2010), and abscisic acid (ABA) (Hauser et al., 2011; Contreras-Cornejo et al., 2015a; Contreras-Cornejo et al., 2015b) aid in plant growth, and control fungal invasion and insect attacks. Moreover, these regulators (e.g., SA) are essential for plant health survival under phytopathogenic attacks, which involve systemic acquired resistance (SAR) and induced systemic resistance (ISR) (Janda and Ruelland, 2015). In addition, JA stimulates plant protection against a wide range of plant pathogens and pests (Kunkel et al., 1993), but is downregulated by biotic stress (Kilaru et al., 2007; Tamaoki et al., 2013) (**Figure 1**). Minor metabolites (e.g., methyl salicylate, pipecolic acid, abietane diterpenoid dehydroabietic, and a glycerol-3-phosphate-dependent factor) have emerged as inducers during the initiation of SAR. These components have been linked to prime SAR activation under biotic exposure (Ryals et al., 1996; Shah et al., 2014). ISR is another defensive strategy defined by activating pathogenesis-related (PR) genes responsible for the secretion of certain defense enzymes. These defensive proteins reduce cell damage caused by free radicals and protect the plant cell wall against destructive fungal diseases (Mwaniki et al., 2005; Yao and Tian, 2005; Reise and Waller, 2009; Derksen et al., 2013).

Furthermore, ISR is related to methyl jasmonate (MeJA) inducible cell wall biosynthesis and lignification (Cheong and Choi, 2003; Yang et al., 2022). In addition, IAA, the primary auxin in higher plants, significantly influences plant developmental stages when in the form of free IAA, compared to IAA conjugates which were primarily ineffective (Zhao, 2010). For example, IAA stimulates the development of expansins, proteins that promote cell wall loosening. As a result, the host is more susceptible to different pathogen invasions. Hemibiotrophic or necrotrophic fungi synthesize IAA and can regulate plant growth to provide nutrition for fungal development and colonization, disrupting plant defensive responses such as programmed cell death (PCD) (Ludwig-Müller, 2015). Studies by Tanaka et al. (2011) and Li et al. (2013) have observed that the pathogen *Magnaporthe oryzae* can release IAA at the site of hyphal infection. In turn, this also stimulates the host to synthesize endogenous IAA at these sites. However, it remains unclear how *M. oryzae* induces host IAA production at the molecular level (Tanaka et al., 2011; Li et al., 2013).

### 2.2 Antifungal metabolites from plants

Antifungal compounds secreted from host plants are categorized into two potential defense groups: The first is called phytoanticipins, and are constitutively synthesized in healthy plants under natural conditions. For instance, *Hebe cupressoides* and blackcurrant (*Ribes nigrum*) produce the phytoanticipin flavanone sakuranetin (Atkinson and Blakeman, 1982) in the leaves of rice (*Oryza sativa*) (Kodama et al., 1992; Perry and Foster, 1994). The second group is synthesized *de novo* in response to biotic (pathogens) or abiotic stressors (e.g., salinization, drought, and heavy metal ion exposure). These SMs may be generated in one organ while being constitutively utilized in another. For example, phytocassanes, a type of phytoanticipin, include flavonoids and phenolics (e.g., coumarins and lignans) (Crozier et al., 2008). The leaves and skin of fruits contain an abundant source of these chemicals. Their precursors play a crucial role in protecting plants under biotic conditions and stimulating nodule formation for biological nitrogen-fixing (Crozier et al., 2006). Phenolic components are plant substances of the phosphate and phenylpropanoid pathways. These materials interact with membrane proteins, causing structural and functional deformation and modifying microbial cell permeability (Küçük et al., 2007). These changes affect membrane dysfunction and subsequent disruption through different methods: (1) interference with the cell energy (ATP), (2) inhibition of the enzyme activity, and (3) prevention of energy consumption (De Oliveira et al., 2011). Plant hosts inhibit pathogen proliferation by secreting different antimicrobial compounds, such as saponins (e.g., Avenacin). For instance, the tomato saponin, tomatine, stimulates phosphotyrosine kinase and monomeric G-protein signaling pathways in *F. oxysporum* cells, resulting in elevated Ca^2+^ and reactive oxygen species (ROS) burst *via* cell membrane binding, causing cell component leakage (Ito et al., 2007). In addition, maize plants infected with *Fusarium graminearum* demonstrated higher levels of sesquiterpenoid phytoalexins and zealexins. Notably, zealexin displayed antifungal efficacy against numerous phytopathogenic fungi (*F. graminearum*, *A. flavus*, *Rhizopus microsporus*) (Huffaker et al., 2011). Plant species generate specific saponins against different phytopathogenic fungi (Osbourn, 1996). Generally, mutualistic or pathogenic interactions between fungi and plants involve the simultaneous molecular signals generated from the interacting species (Pusztahelyi et al., 2015). Recent studies have shown that the Neofusicoccum genus comprises 50 species naturally associated with plants worldwide. Still, many Neofusicoccum species belonging to the Botryosphaeriaceae and Botryosphaeriales families cause diverse grapevine diseases, such as leaf spots, fruit rots, shoot dieback, and vascular discoloration of the wood. Furthermore, diverse SMs were identified from the Neofusicoccum species *N. botryoisocoumarin* A, and *N*. *botryosphaerones* that cause fruit ripe rot harvest disease. Of these, the SM isosclerone, is likely related to both fungal pathogenicity and virulence (Salvatore and Andolfi, 2021). In addition, some SM gene clusters are expressed in the host plant, and others are not. For instance, when *Fusarium fujikuroi* infects another plant, its ability to transcribe *fusaric acid biosynthesis* (*FUB*) 1, which codes a polyketide synthase (PKS) involved in manufacturing the fusaric acid toxin, is inhibited (Niehaus et al., 2014). Thus, it is challenging to predict how SMs will contribute to pathogenesis. For example, the pathogenesis of *Pyricularia oryzae*, which cause rice blast disease, is dependent on the accumulation of fungal melanin as an SM. However, tenuazonic acid, a hybrid non-ribosomal peptide synthetase (NRP)/PKS mycotoxin, nectriapyrones, phytotoxic polyketide compounds, and pyriculols do not play significant roles (Motoyama et al., 2021). Many fungal SM clusters cannot be created in a laboratory setting due to the inability to replicate the specific environmental conditions that typically induce synthesis in nature. Still, their functions can be investigated by selectively activating them. Because specific plant SMs can be hazardous to the producer, the accumulation of these substances is controlled in suitable compartments andoften accumulate in lesser amounts than primary metabolites (Dewick, 2002). Nevertheless, in specific tissues, they can accumulate to greater concentrations (Takanashi et al., 2012).

### 2.3 Metabolites in plant mutualistic interactions

Plants actively influence microbial populations that occupy their surfaces or colonize their internal structures (Bednarek et al., 2010). Host plants exude various substances to communicate with the microbial community. A well-studied example is a release of root exudates (e.g., phenolics, amino acids, terpenoids, and sugars) by the host to communicate with rhizosphere microbial species (e.g., arbuscular mycorrhiza (AM)) (Baetz and Martinoia, 2014). The relative abundance of these chemicals varies according to plant species, genotypes, developmental stages, and environmental conditions (Korenblum et al., 2020). Plant secondary metabolites (PSMs) are divided into non-volatile chemicals and volatile organic molecules (VOCs). These components are secreted *via* plant roots (root exudates) into the rhizospheric zone, actively using ATP as an energy source or passively through diffusion. Root components that influence plant microbiome signal interactions in the rhizosphere are becoming more widely understood (Sasse et al., 2018; Yuan et al., 2018; Williams and de Vries, 2020). Interestingly, it has been suggested that various rhizodeposits may affect the rhizosphere microbiome community (Pascale et al., 2020) and that some root exudates influence the microbiome assembly prior to microbial root surface interactions (Sasse et al., 2018). In addition, coumarin, triterpenes, flavonoid, benzoxazinoid, and phytohormones are secondary metabolites that impact both the growth and inhibition of specific rhizospheric microbes (Jia et al., 2016; Holmer et al., 2017; Hu et al., 2018; Voges et al., 2019). These findings will aid in elucidating how natural habitat diversity, crop genetic variation, and plant introduction between locales may manipulate plant microbiome recruitment *via* root exudates (Pang et al., 2021). For example, the legume plant soybean (*Glycine max*) has been investigated for its mutualistic connections with an arbuscular mycorrhizal fungus, which releases a variety of metabolites into the soil (e.g., saponins) (Sugiyama, 2019). Flavanones (such as strigolactones) are signal molecules for soybean-arbuscular mycorrhizal fungi symbioses which can boost ectomycorrhizal fungal growth and improve AM fungi colonization. In addition, flavanones have been shown to stimulate ectomycorrhizal fungal spore germination in the genera *Pisolithus* and *Suillus*, as well as induce synthesis of symbiotic effector protein in the mushroom *Laccaria bicolor* (Garcia et al., 2015; Pei et al., 2020). In line with these findings, the suppression of flavonoid and phenylpropanoid secretion inhibits endophyte and ectomycorrhizal colonization of the maize and poplar tree root system, respectively (Alam et al., 2021).

## 3 Phytopathogenic fungi

Interactions between plants and fungi are complex and elicit many molecular reactions. For example, fungal penetration and infection process often requires several key steps to develop the disease: host-pathogen contact, release of phytotoxins, and cell wall degrading enzymes (CWDEs) for host tissue penetration, initial lesion development in host tissue, lesion enlargement, then eventually, the death of the vulnerable plant (Laluk and Mengiste, 2010). However, the cuticle-covered plant cell wall is the primary line of protection against pathogen invasion. To overcome this barrier, the pathogen must penetrate the plant by either directly penetrating the plant *via* hydrolytic enzyme secretion or indirectly through natural openings such as stomata or physical wounds (Ghozlan et al., 2020).

Meanwhile, incompatible interactions and susceptible plants may initiate various defense signals but similar gene expression patterns. Interestingly, the period required to alter the transcriptome was observed to generate the most variation, whereby defense gene activation required more time in response to a compatible interaction than an incompatible interaction (Li et al., 2006). The ability to generate a phytotoxin to prevent the growth of plants correlates with pathogenicity in various fungal infections. Toxins produced by fungi are either host-selective (HSTs) or non-host-selective (NHSTs). HSTs are often active only against host plants, have a distinct mechanism of action, and are poisonous to the host. Therefore, HST synthesis is critical for the virulence of these fungi (Horbach et al., 2011). HST toxins are chemically varied, ranging from low-molecular-weight molecules to cyclic peptides. The polypeptides required to create HSTs are derived from genes found on a conditionally dispensable chromosome that regulates host-specific pathogenicity (Hatta et al., 2002). The process of host-selective pathogenesis *via* HSTs is well recognized, and around 20 HSTs have been identified. While the earliest stages of the disease do not differ significantly between necrotrophs, hemibiotrophic, and obligate biotrophic fungi, diverse techniques for acquiring nutrients are employed. For example, necrotrophic fungi have a more comprehensive host range than biotrophic fungi (Ghozlan et al., 2020).

### 3.1 Biotrophic fungi

Biotrophic plant fungi are one of the significant causes of crop losses. Successful pathogenesis requires the formation of specialized structures surrounding the host plant plasma membrane, such as haustoria and intracellular hyphae, to facilitate attachment, host recognition, penetration, and proliferation. The regulation of fungal gene expression can interfere with haustorium and hyphae formation, preventing them from forming and reducing overall pathogenicity (Kahmann and Basse, 2001). In addition, biotrophic fungi have evolved a limited secretory activity, particularly of lytic enzymes. Furthermore, carbohydrate-rich and protein-containing interfacial layers separate fungal and plant plasma membranes, resulting in long-term suppression of host defense. Finally, haustoria are used for nutrient absorption and metabolism. Biotrophic fungi have numerous strategies for protecting their effectors from plant receptor molecules.

Once a fungal effector has evaded the mechanism of plant defense, the plant can no longer prevent further infection. Subsequently, the host reduces the production of defense signaling molecules like salicylic acid (Mendgen and Hahn, 2002). In addition, plants use two main strategies to restrict the invasion and growth of biotrophic fungal pathogens: penetration resistance and programmed cell death (PCD)-mediated resistance. The host plant strengthens cell walls and membranes to suppress spore germination and prevent haustorium formation through penetration resistance. In addition, a second resistance mechanism is activated to terminate the nutrient supply of the penetrated epidermal cell, which inhibits fungal growth and induces programmed cell death (Gebrie, 2016).

Moreover, immune defenses induced by biotrophic pathogens involve the accumulation of antimicrobial metabolites and local cell death conferred by a hypersensitive response (Toruño et al., 2016). Plant pathogens use effectors to inhibit immune responses (Toruño et al., 2016). While specific effectors have evolved to circumvent immunological identification by the plant, others defend the fungus from plant-derived apoplastic defensive strategies or modify intracellular plant responses (De Jonge et al., 2010; Marshall et al., 2011; Hemetsberger et al., 2012; Tanaka et al., 2014). Plant defenses are frequently not unique to a particular pathogen but target a broad spectrum of microorganisms. As a result, various pathogens have developed effectors that target the same defensive responses independently from one another (Ökmen and Doehlemann, 2014). In contrast, suppressing defense-related host responses by one pathogen may encourage subsequent infections by other pathogens (Abdullah et al., 2017).

Biotrophic fungi and their respective metabolism have been investigated chiefly on nonobligate biotrophs such as *Cladosporium fulvum* (Thomma et al., 2005), *Mycosphaerella graminicola* (Palmer and Skinner, 2002; Deller et al., 2011), and *Magnaporthe grisea* (Talbot, 2003). Biotrophs generate haustoria for nutrition absorption, prevent the induction of host defense, and alter the host plant metabolism (Panstruga, 2003; Biemelt and Sonnewald, 2006). Little is known about obligatory biotrophs, such as powdery mildews or rust fungi (Duplessis et al., 2011). Species derived from *M. grisea* cause disease in at least 50 grass and sedge plant hosts, including maize, rice, rye, wheat, barley, oats, finger millet, perennial ryegrass, weed, and ornamental grasses (Couch and Kohn, 2002). Within the species complex, *M. oryzae* (previously known as *M. grisea*) isolates form the pathotype *Oryza*, which causes rice blast disease. *M. oryzae* infections typically lose 10-30% of rice production annually. This fungus affects all aerial portions of rice, causing leaf blast, collar rot, neck and panicle rot, and node blast (Skamnioti and Gurr, 2009). Analysis of the rice transcriptome infected with *M.* oryzae indicated that host phytoalexins were highly expressed, suggesting their vital role in response to the pathogen attack. Different gene expression patterns against compatible and incompatible fungal strains have also been observed, suggesting more substantial implications against incompatible interactions. For example, several rice genes have demonstrated roles in the biosynthesis of certain phytocassane: *OsCPS2* and *OsKSL7* in the biosynthesis of phytocassane A-E, and, *OsCPS4* and *OsKSL4*, in the biosynthesis of momilactone A and B, of which showed incompatible upregulation. Furthermore, two days following inoculation, resistant plants accumulated more phytoalexins in a shorter period than susceptible plants (Hasegawa et al., 2010). Nonetheless, significant phytoalexin gene induction was detected in the incompatible interaction compared to gene expressions in the compatible interaction in the same rice cultivar at the beginning of the infection phase (Kawahara et al., 2012).

### 3.2 Special metabolites of biotrophic phytopathogens

Although the mechanistic understanding of the obligate biotrophic filamentous pathogenic effectors remains understudied, numerous facultative biotrophs and necrotrophs have been functionally described. Moreover, insufficient genetic transformation and gene deletion tools available to perform reverse genetics in obligate biotrophs are significant factors in this knowledge gap (Ökmen et al., 2021). Various methodologies are used to characterize the functional properties of obligatory biotrophic pathogen effectors. One such approach is the heterologous expression of pathogenic effectors in plants (Germain et al., 2018; Xu et al., 2020). However, heterologous expression of specific effectors in planta can cause significant pleiotropic abnormalities that compromise symptom assessment. For example, the type III secretion system (T3SS) of *Pseudomonas syringae* and *Pseudomonas fluorescens* or *Pseudomonas atropurpurea* is employed in another approach for functional evaluation of numerous intracellular effectors from obligate biotrophs, such as rusts and *powdery mildews*. The growth of *Pseudomonas* spp. transformants that transport the required effectors into the host cell during infection positively contribute to virulence (Upadhyaya et al., 2014; Ramachandran et al., 2017; Montenegro Alonso et al., 2020).

Although the T3SS system transports effectors into host plant cells, it is unclear whether fungal effectors, which function in the apoplast and are essential for haustorium development, play a role in host colonization (Ökmen et al., 2021). Furthermore, several biotrophic pathogen effectors decrease PAMP-triggered immunity (PTI) and enhance disease establishment. Because PAMPs from bacterial and fungal pathogens differ due to phylogenetic distance, PTI responses elicited by *Pseudomonas* spp. cannot be well-evaded by pathogen effectors unless the signaling pathways that lead to PTI are conserved (Ökmen et al., 2021). In addition, a host-induced gene silencing (HIGS) test was established to assess the virulence function of obligatory biotroph effector genes during host colonization (Yin and Hulbert, 2018; Yang et al., 2020). However, the requirements for stable transgenic host lines of HIGS constructs make this technique time-consuming (Ökmen et al., 2021).

Chemical signals are required for *M. grisea* appressorium development. The appressorium of *M. grisea* adheres tightly to the host surface by synthesizing an appressorial glue made of glycoproteins, neutral lipids, and glycolipids (Gilbert et al., 1996). The nontoxic plant metabolite zosteric acid binds water and improves the surface’s hydrophilicity, reducing the appressorial glue’s binding ability. Zosteric acid prevents *M. grisea* spore adherence and infection capability on artificial hydrophobic surfaces and plant leaves (Todd et al., 1993; Stanley et al., 2002). Two effective inducers of germination and appressorium development in *M. grisea* are 1,16-hexadecanedial and 1,16-hexadecanediol, derived from cutin monomers (Gilbert et al., 1996). In order to penetrate the rice cuticle, turgor pressure inside the appressorium increases up to 8MPa, resulting in several biochemical and morphogenetic changes (Howard et al., 1991). This unusually high pressure and mechanical penetration raised questions about the role of secreted fungal cell wall-degrading enzymes in the initial stages of natural host invasion (Howard and Valent, 1996). In addition, a thick melanin layer was observed to be deposited outside the cell wall of *M. grisea*to to achieve the observed high turgor pressure. Several natural substances reduced melanin synthesis safely to prevent dangerously high pressure, likely by targeting the same sites. These include coumarin (phenolic substances), a typical plant SM, scytalol D synthesized by Scytalidium spp., and the lipid biosynthesis inhibitor cerulenin (Wheeler and Bell, 1988; Thines et al., 1995), initially derived from an isolate known as *Cephalosporium caerulens*, related to the phytopathogenic fungus *Sarocladium oryzae* found in rice (Bills et al., 2004).

## 4 Fungal secondary metabolites

Research has begun to uncover the function of secondary metabolites from fungi, including polyketides, terpenes, and nonribosomal peptides (Boruta, 2018). Although these secondary metabolites have no direct role in growth, development, reproduction, or energy production, there is some indication of a positive role in regulating host survival in different ecological niches. For example, most secondary metabolites exhibit biological activity, encouraging efforts to produce new drugs (Uzuner et al., 2012). Penicillin is the first antibiotic with a broad spectrum of activity, the most well-known secondary metabolite of a fungal origin. The discovery of penicillin altered medical practices, redirected pharmaceutical research and undoubtedly saved many lives during World War II. The source of this antibiotic was isolated from the fungus *Penicillium notatum*, by Alexander Fleming. This discovery marked a significant advancement in medicine and science for treating bacterial illnesses (Bennett and Chung, 2001). Inhibiting the growth of fungi and preventing systemic resistance mechanisms are often carried out by secondary metabolites found in thousands of fungi (Keller et al., 2005). Terpenoids, polyketides, nonribosomal peptides, and compounds produced from shikimic acid make up the four major chemical categories of fungal secondary metabolites.

The microbial fermentation process is the most frequently utilized technique in microbial biotechnology. The primary industrial applications of this technology include the detection of secondary metabolites and the production of certain molecular products. In liquid-grown crops, fermentation occurs under submerged conditions (Kour et al., 2020). Solid-state conditions are one type of technology for cultivating microbial culture processes to generate bioactive compounds in agro-industrial settings. A vital feature of this technology is that fermentation occurs on a solid substrate with low moisture content (lowest limit: 12%). Since fungi can grow in lower water levels than bacteria, which need free water for fermentation, they are suitable for this culture method. Solid-state fermentation is a low-cost method based on organic materials like wood chips and agricultural waste. It has been employed for synthesizing both primary and secondary metabolites because of its advantages over submerged fermentation (Kellogg and Raja, 2017). The biological consequences of secondary metabolites, particularly their antibacterial activity, have recently become known. *Alternaria alternata*, *Alternaria brassicicola*, and *Aspergillus fumigate* have antifungal, antibacterial, anthelmintic, and anticancer activities. In addition, there are known uses for fungal secondary metabolites in many different industries, including agriculture. Population growth has resulted in a discernible rise in agricultural demand (Akone et al., 2016). A key concern is the increased demand for food crops to satisfy consumer demand. Using fungal secondary metabolites, which are aimed at improving the efficient growth of food crops, is one of many strategies to boost crop productivity. In addition, fungi and insects that aid in pests (such as aphids, beetles, and grasshoppers) and pathogen control are targeted by fungal secondary metabolites. Numerous reports have suggested that these secondary metabolites, which were screened from different fungal species, have agricultural uses (Almassi et al., 1991).

Throughout history, in both ancient and modern times, fungi have been widely employed in food and medicine. Alcohol, antibiotics, enzymes, chemical compounds, and medication can be produced from fungi. Thus, the use of fungi in the production of diverse proteins further emphasizes their importance. For example, fungi produce anticancer, antidiarrheal, antitumor, antihypertensive, and antimutagenic medications with a significant impact on the global growth of nutraceuticals and the functional food business. The fruiting body of the mushroom, its mycelium, and its extracts or concentrates have the potential to treat a wide range of human illnesses and are, therefore, recognized as functional foods. However, there are still numerous instances where the active principle must be discovered to comprehend the mechanism fully. Fungi produce various chemicals, from fundamental metabolites such as enzymes and citric acid to secondary metabolites like antibiotics and alkaloids. The five fungal secondary metabolic pathways include polysaccharides, amino acids, the polyketide pathway, the mevalonic acid pathway, and the shikimic acid pathway. Most of these components are employed as medicines (Shu, 2007). Some of these include the fungus *Agaricus campestris* to treat sinusitis, inflammation, and tuberculosis, *Laricifomes officinalis* as an herbal remedy to cure night sweats and diarrhea, andfinally *Daedaleopsis flavida*, which effectively treats jaundice by lowering bilirubin and biliverdin levels (Devi et al., 2020).

### 4.1 Arbuscular mycorrhizal fungi

It is well known that Arbuscular mycorrhizal fungi (AMF) increase the availability of nutrients and water uptake for host plants, resulting in 4-25% of their photosynthetic energy toward AMF roots (Wipf et al., 2019). As obligate biotrophs, AMF soil microorganisms cannot complete their life cycle without colonizing host plants. Sugar transport constitutes a key pillar for understanding this behavior. Both partners exert strict control over the flow of carbon produced during photosynthesis in plant leaves toward the root that AMF has infected. Starch was once considered a crucial component for AMF symbiosis (Berdeni et al., 2018; Chen M. et al., 2018), since sucrose is derived from the host and is broken into functional hexose forms for the fungal symbiont. Therefore, AMF exclusively consumes hexose from its host. However, due to differences in structure and activities, AMF components play diverse roles in hexose metabolism (**Figure 1**). For example, AMF extra radical mycelium lacks the sucrose-cleaving capacity that is necessary for it to be able to digest hexoses and improves photosynthesis (Gupta et al., 2021). Phloem transports sucrose to the apoplast, where invertases and synthase break it down before being released into the cortical cell of the root system to be supplied to AM fungus (Wang et al., 2017).

Additionally, some studies indicate that AMF colonization increases alkaline invertase activity, making hexose accessible for AMF development. After being taken up by the fungus from the host plant, carbon is transferred to different radical mycelium to generate spore and hyphal growth. Signaling pathways play critical roles in environmental sensing, mating behaviors, morphogenesis, and host communication in plant pathogenic fungi. Ca^2+^ has a crucial role as a messenger molecule in the signaling process for various processes (Liao et al., 2018). Host constitutive defense includes chemical and physical barriers that prevent the majority of microorganisms from attaching to or entering the plants (Paludan et al., 2021). In plant-pathogen interactions, the first layer of induced defense is the pathogen-associated molecular pattern (PAMP)-triggered immunity (PTI). Defense mechanisms are triggered when pathogen-derived chemicals or structures are recognized by plant pattern recognition receptors (PRRs), inducing PTI (Cavaco et al., 2021). Pathogens send effector proteins into host cells, where they disrupt defensive mechanisms to evade PTI. Plants recognize effectors *via* resistance (*R*) genes, resulting in effector-triggered immunity (ETI), which improves and accelerates the immune response. When one or more pathogen effectors successfully obstruct PTI responses, pathogens can infect susceptible hosts (ETS) (Thakur et al., 2019; Naveed et al., 2020). There are many changes between ETS and ETI due to the co-evolution of effectors in pathogens and corresponding *R*-genes in the host plants. This network of interconnected pathways frequently overlaps and results in a single phenotypic outcome (Abro et al., 2019). PTI and ETI are considered two separate and succeeding branches of plant immunity, but there are several situations where effectors are qualified to be categorized as PAMP effectors. Exogenous elicitors include the pathogen membrane or cell wall components, host enzymes, or the pathogen itself, recognized through the undermining of host defenses and/or the facilitation of nutrition intake (Thakur and Sohal, 2013; Meena et al., 2022). Endogenous elicitors are chemicals that originate from host plants and are often the outcome of damage caused by pathogen enzyme activity. Consequently, these PAMPs and damage-associated molecular patterns (DAMPs), synonymous with exogenous and endogenous elicitors, induce PTI. As discussed below, PAMPs can have various chemical structures, ranging from proteins to carbohydrates (**Figure 1**).

### 4.2 Genetic background of the fungal SMs production

Fungal SMs are typically generated from the activation of biosynthetic gene clusters (BGCs), comprised of synthases and/or synthetases (such as polyketide synthase, terpene synthase and/or cyclase, non-ribosomal synthase, and isocyanide synthase) that utilize primary metabolites as substrates to create carbon backbones, which are further modified by tailoring enzymes (Clevenger et al., 2017). Some BGCs contain cluster-specific transcription factors that positively control the activity of the other BGC genes and regulatory genes that encode proteins to reduce the toxicity of the BGC being produced. Certain enzymes massively polymerize primary metabolites during the formation of SMs to synthesize the backbone of the SM compound. Constituents are then added to change SM bioactivity. The enzymes responsible for backbone synthesis also determine the type of chemically generated SM formed (Monroy et al., 2017). For instance, non-ribosomal peptide synthetases (NRPSs) from acyl-CoAs make polyketides and terpene synthases. Cyclases manufacture terpenes from activated isoprene units and non-ribosomal peptides from amino acids, respectively. Caution should be exercised when assuming the sources of lipopeptides since the term is also used to describe compounds made from peptides produced by ribosomes coupled to fatty acids or isoprenoids. Many people refer to the PKS-NRPS-derived hybrid secondary metabolites as lipopeptides (Grgurina, 2002; Cochrane and Vederas, 2016). Since these small signal molecules influence bacterial-fungal interactions, the underlying mechanism beyond the regulation of antagonistic activity must be explored further. For example, *Ralstonia solanacearum* causes bacterial wilt in many plants that can also produce molecules such as ralstonins, isoquinolines, and lipopeptides. Contrastingly, these molecules are involved in the growth inhibition of the pathogen *Aspergillus flavus* by BGC downregulation (Khalid et al., 2018). It is suggested that these molecules affect microbial diversity, pathogenicity, and microbial survival.

#### 4.2.1 Environmental genes

The environment strongly influences the developmental stage at which fungi begin to generate SMs. Environmentally-sensitive fungal genes can activate BGCs both transcriptionally and epigenetically (Hagiwara et al., 2017). The “one strain-many compounds” (OSMAC) strategy for mining metabolites reflects the long-withstanding concept of the significance of dietary input. For instance, early studies on aflatoxin formation uncovered that temperature and light could promote or delay the production of natural compounds (Cary et al., 2015). Numerous research teams have linked the production of fungus secondary metabolites to red and blue light photoreceptors and/or their associated signal transduction pathways. The aflatoxin and associated sterigmatocystin mycotoxin BGCs are a well-known cluster inhibited by white light (Nazari et al., 2016). Natural products like the polyketide dihydrotrichotetron can be controlled by known transcription factors like *creA* and/or *cre1* genes. The Velvet complex component *veA*, the blue light receptor FphA, and the phytochrome FphA of the organism *Aspergillus nidulans* form a complex that has been used to explain the connection between light perception and the production of SMs (Kenne et al., 2018). The revelation that phytochrome FphA of *A. nidulans* connects with both blue light receptors and VeA, provides a molecular explanation for the relationship between light sensing and the generation of secondary metabolites (Larkin et al., 2018). The transcriptional responses of BGCs to altered environmental stress pathways, particularly oxidative stress, follow the theory that SMs, like aflatoxin, function as reactive oxygen species inhibitors (Zhao et al., 2017).

#### 4.2.2 Transcriptional regulation

Up to 50% of fungal BGC promoters possess palindromic patterns recognized by a cluster-specific transcription factor, usually a C6-zinc cluster protein (Brown et al., 2015). A single BGC may occasionally contain many transcription factors binding sites. However, it is now known that various C6 transcription factors can regulate genes within BGCs and various metabolic pathways. This contrasts with the previous belief that C6 transcription factors exclusively control genes inside a single BGC. Numerous “broad-domain” transcription factors involve positive and negative regulation of many BGCs, such as the pH regulator PacC, the nitrogen regulator AreA, and the CAAX basic leucine zipper protein HapX47 (Amaike and Keller, 2009). The Velvet complex, however, is a vital transcriptional complex that influences the overall regulation of secondary metabolites in all fungal taxa examined up to this point (Pettit, 2011). Follow-up studies using microarray and RNA-seq to analyze cascades of regulators involved in the production of secondary metabolites have provided insight into how BGC is controlled. BrlA, a C2H2 transcription factor, is required to produce conidiophores by *Aspergillus* and *Penicillium* species. In addition, genetic, ecological, and mechanistic studies have provided irrefutable evidence that the fundamental building block of microbial communication is composed of fungal chemicals (Scherlach and Hertweck, 2018). The regulatory networks of secondary metabolites and the epigenome have been linked in numerous studies. For example, the sterigmatocystin BGC of *A. nidulans* contains methylation marks on histone H3 residues during the active growth phase but is silent during the early growth phase (Schüller et al., 2022). Although this study provides limited evidence that fungal SMs improve ecological fitness (Alam et al., 2021), further research is essential to confirm similar phenomena. Together, it is likely that many SM-encoding genes are controlled in a manner that is consistent with fungal growth or in response to biotic and abiotic stressors. Moreover,loss or overproduction of specific SMs can affect alter fungal development and the ability to interact with fungi of the same and/or other kingdoms (Kalinina et al., 2017).

## 5 Do fungi have self-protection?

In order to prevent self-harm from hazardous BGC products, BGCs can encode efflux transporters and cellular BGC intermediate transporters. (Kaaniche et al., 2019). Numerous fungi (e.g., *Agaricus campestris*) heal inflammation, sinusitis, and tuberculosis and have been used in traditional medicinal practices. *Laricifomes officinalis*, an herbal remedy, can treat diarrhea and night sweats (Saini et al., 2016). With all these therapeutic and physiological benefits, additional scientific proof is needed to support these results (Venkatesh and Keller, 2019). Mushrooms are highly valued in the food and pharmaceutical industries. They include more than simply fundamental chemicals, which might offer certain advantages. Maintaining the quality of mushrooms is difficult, though, because even small changes to their genetics, soil, moisture, temperature, and harvesting period can significantly impact the concentration of vital components.

### 5.1 Manipulation of programmed cell death

Programmed cell death (PCD) is apparent in all biological systems and present in most evolutionary lineages (Frank and Vince, 2019). The widespread presence of PCD is likely derived from an ancient origin or that it is a result of extensive evolution. In either scenario, PCD is crucial to the life histories of many species, including fungi (Ramsdale, 2008). Different types of cell death mechanisms can reveal the underlying variation in the cell biology of subject species. However, it is now understood that similar molecules to SMs play a crucial role in the induction of cell death in organisms such as bacteria, yeast, plants, worms, flies, and even humans (Carmona-Gutierrez et al., 2010). Numerous studies have focused on elucidating the relevance of similarly functioning molecules across these different organisms with the possibility of opening a new avenue of research to produce drugs to combat infectious diseases that affect plants and animals. The primary hallmarks of PCD reactions in animals are a series of morphological and biochemical abnormalities that are mediated by external (extrinsic) or internal (intrinsic) cell suicide programs (Green and Llambi, 2015).

Death signals in the intrinsic death pathway cause the release of mitochondrial proteins, which amplifies the caspase cascade (Tang et al., 2019; Kist and Vucic, 2021). Signals transmitted by death receptors that are a part of the tumor necrosis factor (TNF) receptor superfamily directly initiate the caspase cascades extrinsic route. Activating caspase-independent suicide pathways also include the movement of the apoptosis-inducing factor (AIF) from the mitochondrion to the nucleus. This presents another way stress can lead to cell death. DNA degradation and nuclear fragmentation, which are brought on by various nucleases, are typically linked to PCD. In addition, the loss of phospholipid asymmetry caused by phosphatidylserine moving to the plasma membrane outer leaflet is another feature of PCD. Many fungi now exhibit programmed cell death responses, although most research has concentrated on yeasts. Functional studies of yeast genes show some similarities at the molecular level between apoptosis in higher eukaryotes and apoptosis in fungi. This is primarily due to the discovery of caspase-like cysteine protease homologs (Bhattacharjee and Mishra, 2020). Since many known elements of higher plant and animal apoptosis have been identified in fungal genomes, it is predicted that the proteins responsible for fungal cell death will differ substantially from their mammalian counterparts to allow for pharmacological treatments (Camilli et al., 2021; Williams et al., 2021). The possibility that novel antifungal drugs and fungicides will be developed to eliminate infections by inducing fungal cell suicide rises with the finding of PCD responses in a model pathogenic fungus. Identifying the endogenous molecular switches that start fungal death is essential to achieving these objectives (Dyer, 2019).

The fundamental cellular similarities between pathogenic fungi and their hosts make it challenging to develop and administer effective antifungal/fungicide therapy. However, long-term use is not recommended because many antifungal medications are incredibly harmful (Jorgensen et al., 2017). Antifungal drugs currently concentrate on vital fungal cell surface activities, including plasma membrane or cell wall biogenesis. Various antifungal drug classes are currently available to treat clinical fungal infections. Fungicides are also often used to treat various plant diseases, targeting both general and specific fungal diseases (Pegorie et al., 2017). Two in particular, include sulfur-containing fungicides and strobilurins, which block the activity of the ubiquinol-cytochrome oxidoreductase of the cytochrome bc1 complex I. Finding new targets for antifungal therapy is crucial since pathogenic fungi routinely display dangerously high levels of resistance to conventional antifungal medications in clinical settings, especially those targeting the electron transport chain (Bongomin et al., 2017). Several variables, including the patient’s pharmacokinetics of medication and the state of their immune system, can influence the effectiveness of antifungal therapy. For example, resistance may emerge due to ineffective patient dosing, inadequate antifungal drug selection, or repetitive exposure (Naderer and Fulcher, 2018).

A decrease in ATP levels often accompanies cell death, although cell fate also depends on plasma membrane integrity and mitochondrial membrane potential (Gonçalves et al., 2019). Only a few environmental factors, such as acetic acid, hydrogen peroxide, and AmB, can cause *C. Albicans* to initiate a PCD response, characterized by the rapid emergence of several traditional apoptotic markers seen in mammalian cells. This includes a loss of cell viability when stained with dye propidium iodide, sustained oxygen consumption, and metabolic activity during cell death, and the production of ROS in apoptotic cells (Nguyen et al., 2018). The overexpression of plant defense molecules results in a hyperbranched phenotype or spiral hyphae production. Distinct hyphal branching patterns have long been linked to growth suppression.

Interestingly, proteins involved in the PCD response of filamentous fungi possess homologous more similar to those in the mammalian cell death pathway than in *Saccharomyces cerevisiae*. Additionally, some proteins linked to cell death in humans and filamentous fungi share a higher degree of similarity when compared to yeast and filamentous fungal species (Man et al., 2017). These two characteristics make filamentous fungi, particularly appealing to investigate PCD, in addition to the fact that many of them have been widely employed as model organisms for cell biology.

### 5.2 Recognition of pathogenic substances necessary to initiate defense response

An infection may be intracellular or extracellular, depending on the organism. Bacterial pathogens and other parasites can grow intracellularly or extracellularly; however, all viruses infect cells and do so intracellularly. The innate immune system must recognize the external pathogen or infected host cells to respond effectively (Ádám et al., 2018). The immune system continuously scans the organism for signals of infections. Host immune cells and/or molecules are dispatched to the location of infection when signs of a pathogen attack are detected. These immune factors can recognize the type of disease, promote clearing the disease or pathogen, and then turn off the immune response once the infection has been eradicated to prevent unnecessary harm to host cells. Secondary exposure to the same pathogen can result in the immune system utilizing these same immune factors to respond more effectively (Chen L. et al., 2018). Mobile immune sensing and focused immune systems are absent in plants. However, in response to the invasion of potentially phytopathogenic organisms, they can generate similar, long-lasting memory of the pathogen attack and activate efficient defense mechanisms of innate immunity. The plant defense system utilizes a range of immunological sensors in nearly every plant cell that may detect pathogens and initiate an immune response, as well as the ability of defense-related messages to travel long distances through the phloem (De Lorenzo et al., 2018). The innate immune system of plants detects the presence of potentially harmful organisms *via* the appearance of certain chemicals that act as warning signs. Pathogens or plants themselves may be the source of these chemicals. These chemicals are identified by immunological sensor receptors, which, when engaged, induce defensive signaling responses. Throughout their lifetime, plants interact with a variety of bacteria. These interactions can be advantageous or detrimental, leading to mutualistic or pathogenic linkages (Rodriguez-Moreno et al., 2018). Plants can alter their innate immune system to mount an appropriate response to various helpful and harmful germs by changing the mechanism triggered by the bacteria. Generally, plants can detect ‘non-host’-derived cells and molecules and stop microorganisms at the front line upon initial recognition. The effective plant immune system must clear infections that circumvent these primary barriers to prevent the spread of microbial invasion. Plants lack the somatic adaptive immune system and mobile defense cells in mammals. Instead, they rely on each cells ability to exert innate immunity, with sick cells capable of delivering systemic signals and plant cells with the capacity to recall previous infections. The first branch of defense uses plant cell-surface-anchored microbe, pathogen, or damage-associated molecular pattern recognition receptors (PRRs) to induce a variety of reactions, including MAMP-triggered immunity (MTI), PAMP-triggered immunity (PTI), and DAMP-triggered immunity (Sun et al., 2018). The second branch recognizes microbial effectors, the virulence factors that prevent MTI, and initiates effector-triggered immunity using resistance (R) proteins (ETI). When these immune responses are activated, a complex series of signaling events that block pathogen attacks are triggered. Each plant species has a natural ability to recognize, react to, and prevent other potential diseases (Walley et al., 2018). This concept has resulted in a plethora of research examining how plant signals promote the growth of diseases in various plants (Underwood, 2012). Studies on the gene expression of the hemibiotrophic fungus *Colletotrichum higginsianum* spores have shown the influence of host plant signals on early pathogen growth (Yan, et al., 2018).

## 6 *Trichoderma* applications in the agricultural sector

**Figure 2:**
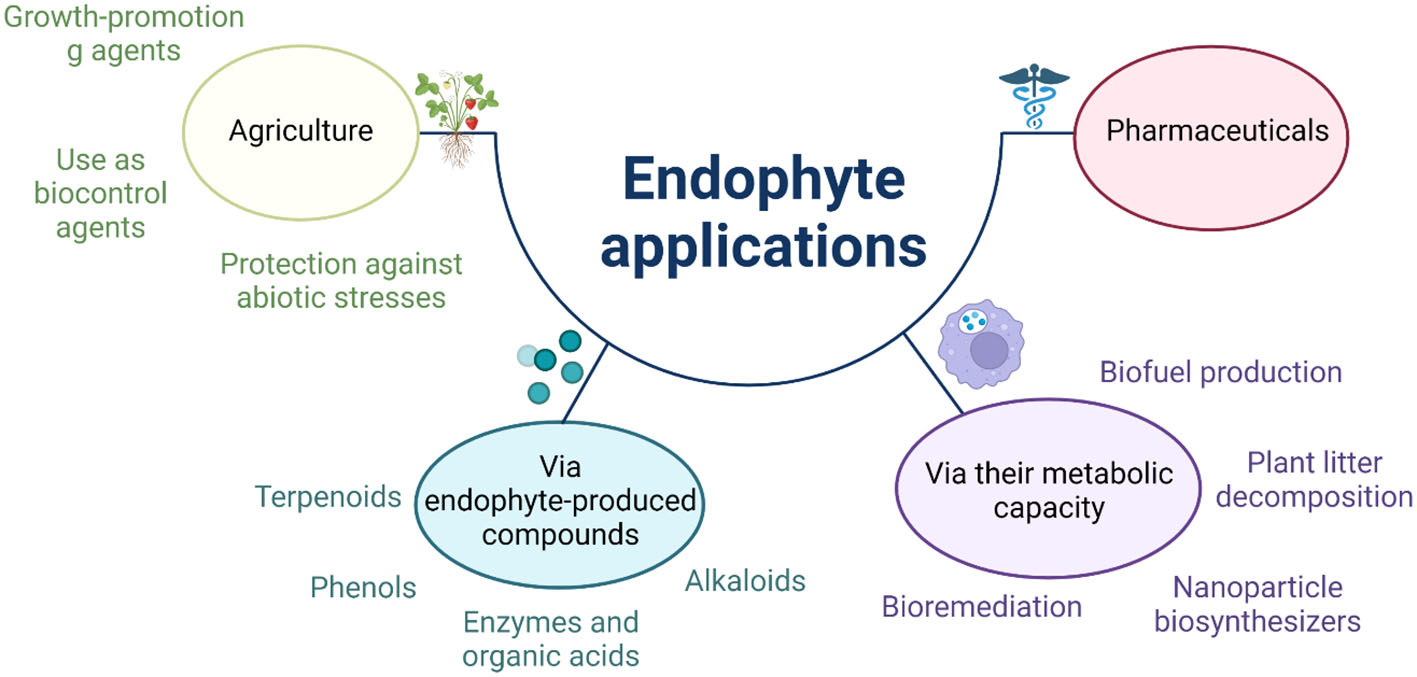
Schematic shows the fungal bioinoculants) applications in response to diverse stress conditions.

Endophytic fungi are a particular class of microorganisms living in symbiotic/mutualistic relationships with plants without causing disease symptoms. These microbes have adapted to the surrounding environment through genetic biodiversity and eventually uptake the plant host DNA. During the process of evolution, plants migrated habitats from aquatic to terrestrial environments, which were impacted by high carbon dioxide, low soil nutrients, and temperature and water availability changes. Endophytes may cause significant and profound shifts in agricultural practices. Beneficial bacterial and fungal plant endophytes are used to boost growth, serve as biocontrols, suppress disease, and mediate stress tolerance as an eco-friendly strategy for agricultural management. This section presents how they are used in various agricultural contexts, focusing on members of the *Trichoderma* species (**Figure 2**). Since the 1960s, it has been recognized that *Trichoderma* species can attack other fungi (Klein and Eveleigh, 2002; Contreras-Cornejo et al., 2015).

Additionally, researchers view *Trichoderma* as a possible biological control agent (Contreras-Cornejo et al., 2014; Sood et al., 2020; Ferreira and Musumeci, 2021; Poveda, 2021). Studies have suggested that *Trichoderma* spp. can aid in plant disease resistance, development, and growth (Benítez et al., 2004; Poveda et al., 2020). In addition, *Trichoderma *spp. has demonstrated the ability to detoxify harmful substances and speed the decomposition of organic matter. *Trichoderma *fungi inhabit the soil ecosystem and function as natural decomposers, resulting in their ability to promote growth, improve nutrient uptake efficiency, and ability to alter the rhizosphere. In addition to thriving unfavorable living conditions, *Trichoderma* spp. exhibit virulence trait against plant pathogens (Chaverri et al., 2015). Typically, biological control methods may have little impact on non-target species. However, this does not seem the case with *Trichoderma* spp., after having been classified as a hostile strain after incidences assaulting other pathogens and microorganisms (Podbielska et al., 2020).

Moreover, multiple investigations have shown unexpected impacts of *Trichoderma* species on soil microbial communities. As expected, the structure of the microbial population changes, which is consistent with findings from (Zin and Badaluddin, 2020). As stated by (Cai et al., 2013; Cai et al., 2021), *Trichoderma* spp. have distinct benefits over soil microbes. These fungi may also boost plant development by producing hormone-like compounds to the root and plant growth production. Finally, rapid plant growth promotes microbial populations by exuding vast amounts of root exudates and increasing nutrient availability for microbial use (Imran et al., 2020; Alghuthaymi et al., 2022).

**Figure 3:**
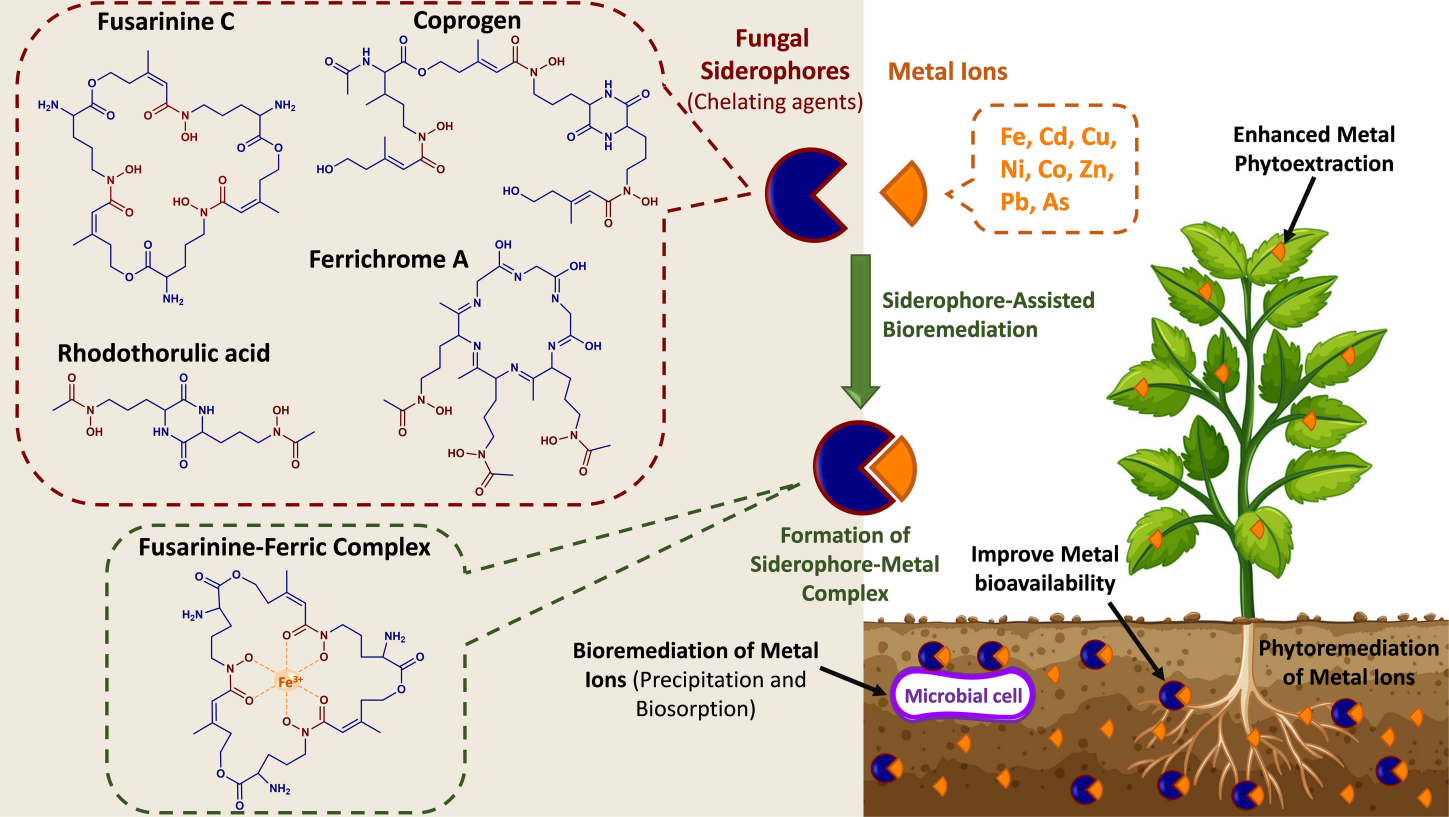
Conceptual representation of the phytoremediation of metal ions by fungal siderophores. The chemical structures of selected fungal siderophores (fusarinine C, ferrichrome A, coprogen, and rhodothorulic acid) and an example of a siderophore-metal complex (Fusarinine C-ferric complex).

*Trichoderma* endophytes are used as biological controls to prime plants against different abiotic stressors such as thermal fluctuation, salinity, low moisture conditions, and resistance to fungicides in soil treatments (Hewedy et al., 2020; Zin and Badaluddin, 2020). These characteristics make *Trichoderma* strong competitors, as they can produce siderophores that release chelated iron (Fe ^3+^) from the surrounding environment to prevent the growth of other fungi as a plant protection mechanism (**Figure 3**). Several molecules from *Trichoderma*, such as pyrones, peptaibols, and terpenes, are secreted as SMs, inhibiting the development of fungi that cause plant diseases. Moreover, fungal mycoparasitism includes locating the host, undergoing morphological changes such as coiling around the hyphal host, forming structures resembling appressoria, and finally penetrating and killing the host (Batool et al., 2020). In addition, these endophytes produce hydrolytic enzymes such as chitinases, glucanases, and proteases to break through the cell wall of the host plant. The synthesis of these enzymes is partially increased due to the induction of diffusible host-derived substances before physical contact with the host. *Trichoderma* spp. are often utilized biocontrol agents against a broad range of root, shoot, and postharvest diseases (Intana et al., 2021). Among roots, *Trichoderma*, is often observed along the root surface and underneath the outermost layer of root cells (Hu et al., 2017). The most common way to apply *Trichoderma* as a biocontrol is at sowing to establish the fungus in and on the plant’s roots. It has been observed that seed coating was an effective method for assuring the colonization of *Trichoderma* spp. on plant roots to protect against pathogens due to their aggressive action on phytopathogens (Gava and Pinto, 2016). For example, using *Trichoderma* species sustains soil health by controlling plant disease growth of pathogens such as *Pythium arrhenomanes*, *Rhizoctonia solani*, *Fusarium oxysporum*, *Alternaria tenuis*, and *Botrytis cinerea*. *Trichoderma* is an endophytic fungus commonly found on leaf tissue, roots, and sapwood and provides several advantages to its host (Cummings et al., 2016). This genus also plays a crucial role in protection against herbivorous arthropods, and critical functions for fungal metabolites in herbivory have also been described (Ramírez-Ordorica et al., 2022). Recently, (Contreras-Cornejo et al., 2018a) investigated how T*. atroviride* affected the ability of maize (Zea mays) to resist the insect herbivore Spodoptera frugiperda. Upon *T. atroviride* inoculation of maize, increased plant growth, decreased herbivory, and altered insect feeding patterns were noted. Increased volatile terpene emission and accumulation of jasmonic acid, which activates defense responses against herbivory, were associated with plant protection. Furthermore, a recent study by (Contreras-Cornejo et al., 2018b) showed that the fungus improved the natural mechanism of parasitism since larvae from maize plants with *T. atroviride* colonized roots were more strongly parasitized than larvae from non-inoculated plants. Another study by (Contreras-Cornejo et al., 2016) revealed that *Trichoderma* could promote systemic resistance and enhance plant nutrient intake in cooperation with plant roots.

Moreover, (Contreras-Cornejo et al., 2021a) demonstrated how *T. harzianum* inoculation could change the arthropod population associated with maize leaves and decrease the prevalence of particular pest insects in open-field conditions. Interestingly, (Contreras-Cornejo et al., 2018a; Contreras‐Cornejo et al., 2021b) introduced new details on the interactions between *T. harzianum* and *P. vetula*, two belowground multitrophic plants, microbes, and arthropods, in the maize rhizosphere that changed how the plant responded to phenotypically and led to the development of root herbivore tolerance. It was also observed that the production of antibiotics or siderophores from several *Trichoderma* secondary metabolites plays a vital role in controlling plant growth and development, as well influencing the growth of plant pathogenic microorganisms in the soil (Contreras-Cornejo et al., 2014; Contreras-Cornejo et al., 2020). Moreover, (Ramírez-Ordorica et al., 2022) revealed that an entomopathogen might have an ecological benefit in that it can accelerate the expansion of the insect population if it can create volatiles that provides olfactory stimulation of egg-laying activity.

## 7 Fungal secondary metabolites-assisted phytoremediation

Aside from assisting plants to cope with biotic/abiotic stresses and promoting their growth, fungal secondary metabolites and enzymes possess vital biological activities applicable to several research fields, including bioremediation and soil health (Hota et al., 2021; Bhadra et al., 2022). It is well known that plant-fungal interactions are dictated directly by soil health. Thus, soil bioremediation, a biological process to remediate environmental/soil pollutants, is essential to promote such interactions and enhance plant growth. The primary sources of pollutants are industrial effluents, sewage water, oil spills, fertilizers, and pesticides. When liberated into the soil, toxic compounds, including heavy metals and complex organic and inorganic compounds, are released, many of which have relatively long half-lives (Khan et al., 2021). Such contaminants represent a significant threat to soil health, leading to degradation and/or permanent destruction of soil and its ecosystem (Borowik et al., 2017; Ding et al., 2022). Soil contaminants may also leach with irrigation and rainwater into the lakes/groundwater, causing water contamination (Khan et al., 2021). Therefore, soil and water contamination has become a global issue that needs to be mitigated due to its direct detrimental effect on both environment and human health. Bioremediation is an emerging green biotechnology approach utilized to revert the contaminated environment to its original state using various biological systems (e.g., microbes, plants) (**Figure 3**) (Dubchak and Bondar, 2019). Phytoremediation is a subcategory of bioremediation in which plants are used to reduce the concentration or toxic effects of contaminants in the environment (Yaashikaa et al., 2022). Although phytoremediation using plants is a cost-efficient and environment-friendly process, elevated contaminants are toxic to plants and can lead to impaired metabolism, reduced plant growth, and slow degradation of soil pollutants (Deng and Cao, 2017; Dubchak and Bondar, 2019). The plant rhizosphere is a natural habitat for microorganisms, including fungi, bacteria, and algae. The microbial plant associations are essential for their host plants, significantly influencing the performance under harsh environments such as heavy metals and toxic compounds.

Both rhizospheric and endophytic microbiota participate in plant health by protecting against pathogenic microbes and improving their productivity. Furthermore, metal resistance and plant growth-promoting microbes are considered one the most efficient and ecologically friendly strategies. Mechanisms to chelate and sequester metals allow fungi to alleviate heavy metal stress. Fungi also play a vital role in element cycling and transforming organic and inorganic compounds and P rock solubility. Thus, fungal metal interactions potentially influence the bioremediation process *via* various metabolic pathways.

Several studies have shown the beneficial fungal interactions with different host plants, e.g., AMF, which support their host plant under heavy metal contamination (i.e., Cu, Cd, Ni, Zn, As, Hg, Pb, Fe, Mn, Co, and Al) by enhancing plant tolerance. Some studies summarized that AMF alleviates heavy metal stress by hindering its uptake by the host plant. For example, AMF decreased the heavy metal impact on Calendula officinalis (pot marigold) development by reducing the uptake of heavy metals (Cd and Pb) and enhancing the beneficial secondary metabolites compared to non-mycorrhizal plants. Similarly, Zn uptake and concentration were reduced in the tomato plants inoculated with mycorrhizal fungi. In addition, heavy metals enter the root cell *via* plasma membrane channels or transporter, impacting plant element distribution and nutrient acquisition *via* different situations, such as detoxification, translocation, and accumulation. Generally, heavy metals are taken up by the roots, located in both the epidermal cells and root hairs, and then translocated to the vascular tissues *via* apoplastic or symplastic pathways (Khalid et al., 2021; Ma et al., 2022). To improve the effectiveness of phytoremediation, endophytic microorganisms have been exploited for their potential to reduce contaminant toxicity to plants and enhance their growth and productivity. Microorganisms have developed several mechanisms to overcome soil metal toxicity, including metal reduction, reduced cell permeability, extracellular sequestration, biosorption, and complexation (Roskova et al., 2022). Their secondary metabolites, known as siderophores, have been shown to play a central role in pollutant elimination and phytoremediation. Siderophores are small molecular weight chelators produced by organisms (e.g., fungi, bacteria, plants) to scavenge Fe from their surrounding environment. Fungal siderophores are of the hydroxamate derived from the non-proteinogenic amino acid ornithine. These hydroxamate-type siderophores have been categorized into four groups based on their chemical structure – fusarinines, ferrichromes, coprogens, and rhodothorulic acid (**Figure 3**) (Rabani et al., 2022). Natural organic chelators are metal-binding compounds that comprise siderophores, organic acid anions, and biosurfactants. Both fungi and plants can release these compounds that scavenge metal ions from sorption sites. Besides their high affinity toward Fe^3+^, siderophores can bind various heavy metal ions, including Cu, Ni, Zn, Cd, Co, and Fe,. (Hofmann et al., 2020). These metals enter microbial cells mainly through diffusion, and their binding to siderophores reduces their concentration in the soil and restricts their release from the cell membrane. Moreover, after their chelation, metals can be sequestered through different extracellular mechanisms, such as biosorption and bioaccumulation (Roskova et al., 2022). Furthermore, by reducing the concentration of toxic metals in soil, siderophore-producing microbes positively contribute to the phytoremediation of toxic metal-polluted soils. Moreover, siderophores serve as redox-cycling compounds, i.e., mediate the synthesis of reactive oxygen species (ROS) and subsequently oxidize organic molecules. This indicates their potential role in the bioremediation of organic contaminants (Gu et al., 2018). In recent years, the potential of microbial siderophores in phytoremediation has gained increasing interest due to their strong affinity to form complexes with toxic metals and their ability to oxidize organic contaminants. The efficiency of phytoremediation may be improved through the inoculation of siderophore-producing microbes or the exogenous application of siderophores to the soil. Various studies reported bacterial siderophores-assisted bioremediation, which can be found elsewhere (Gu et al., 2018; Hazotte et al., 2018; Manzoor et al., 2019; David et al., 2021). However, a few studies highlighted the role of fungal siderophores in the bioremediation of soil contaminants. For example, (Khan et al., 2021) examined the use of the hydroxamate siderophore purified from *A. nidulans* in the bioaccumulation of Cd(II) in *Bacillus subtilis.* They observed increased siderophores-mediated bioaccumulation of Cd (II) by *B. subtilis*, which was siderophore dose-dependent. Here, the optimum siderophore concentration was 50 SU/ml, where higher concentrations negatively affected the *B. subtilis* growth due to the chelation of essential nutrients. A study by (Kumari et al., 2019) examined the use of the hydroxamate-type siderophore (ferricrocin) isolated from *A. nidulans* to reduce the adverse impact of arsenic under toxic conditions on *Triticum aestivum* growth. The formation of the thermodynamically stable ferricrocin–arsenate complex recovered plant growth and assisted in adjusting superoxide dismutase, catalase, and peroxidase activity of wheat while reducing damage caused by lipid peroxidation (Kumari et al., 2019). *Trichoderma*, *Aspergillus*, and AMF were found to enhance phytoremediation of lead (Pd) due to their high immobilizing affinity toward metals through biosorption, insoluble oxalate formation, and/or melanin-like polymer chelation (Schneider et al., 2016). In the study by Bhalerao and Puranik (2007), fungal isolates, *Aspergillus niger*, *Aspergillus terreus, Trichoderma harzianum*, *Cladosporium oxysporum, Phanerochaetechrysosporium, Mucor thermohyalospora, and Fusarium ventricosum*, were evaluated for their ability to degrade endosulfan. *A. niger* was the most tolerant to endosulfan. In addition, some of these fungal strains induced esterification, dehydrogenation, hydroxylation, and deoxygenation during the biotransformation process. Additionally, a few attempts exploited the application of white rot fungi for the bioremediation of organic contaminants. Compared to bacteria, these filamentous fungi provide a myriad of benefits due to their ability to oxide several chemicals and their tolerance to hazardous compounds (Kumar et al., 2022; Rabani et al., 2022). White rot fungi can secrete enzymes that can biotransform organic pollutants into non-/fewer toxic compounds. Interestingly, (Purnomo et al., 2014) investigated the utilization of a white rot fungus, *Pleurotus ostreatus*, in the biodegradation of heptachlor and heptachlor epoxide. They reported 89% and 32% degradation of these two pollutants after 28 days of incubation. Other studies showed that white rot fungi, including *Phlebia acanthocystis*, *Phlebia brevispora*, *Phlebia aurea*, and *Trametes hirsuta* were capable of degrading the organic contaminants dieldrin, aldrin diazinon, and endosulfan (Kamei et al., 2011; Xiao et al., 2011; Bisht et al., 2019). Thus, more studies are needed to investigate the various mechanisms involved in fungal-assisted bioremediation, the potential roles of fungal siderophores, and the effect of contaminants (e.g., toxic metals) on siderophores synthesis. Hence, phytoremediation is the most practicable action to decontaminate serious pollution areas by fungal plant associations to remove heavy metal accumulations, particularly in metal-polluted soils. In addition, the fungal phytoremediation process is a cost-effective and environmentally friendly (without any hazardous effects) technology. However, further research on mechanisms beyond the fungal metal bioremediation/immobilization is needed to employ their efficient use in phytoremediation practices and reduce the harmful impact of toxic metals.

## 8 Conclusion remarks

As the human population expands, food security and safety will become more threatening than ever before. For this reason, it has become imperative that research explore alternative methods to help adopt more sustainable practices to meet the needs of people worldwide. Here, an exciting new avenue for research has been reviewed: fungal secondary metabolites, their origin, biological roles, and current and future applications. Beneficial endophytes such as *Trichoderma* and other fungi are being used as biocontrols, and then circle back to the general knowledge of their having roles in enhancing SAR/ISR among mutualistic interactions with plants. Although this work targets SMs role in improving crop yield and growth during global climate change, it is also important to remember the pharmaceutical and industrial/economic contribution as outlined in (**Figure 2**). For example, antibiotics and their mass-scale production, as well as the synthesis of biofuels.

Additionally, increased production of cellular metabolites to protect plants from disease offers potential for application in biocontrol to reduce significantly synthetic pesticide use, representing an economical and eco-friendly way forward for agricultural sustainability. Besides, the fundamental knowledge governing plant-microbe interactions significantly increased crop yield and food supply. Secondary metabolites play a vital role in plant health by regulating the anti-pathogenic mechanisms in the host plants. Thus, understanding the pathway of SMs and their role in biological control strategies will be essential for improving crop yield and providing novel future green agriculture opportunities. Knowledge of genomic clustering and regulation of SM genes will yield new insight into the evolution of fungal pathogenesis and host defense.

## Author contributions

Conceptualization NAE, OH, AZ, DP; original draft, NAE, OH, AM, OE, AZ , SH, and AE-T. Create and software figures, NAE, OH, AZ, and OE. Review and language editing, OH and AM. All authors approved the submitted version.
